# The Existential Breastfeeding Difficulty Scale’s influences on the caring dialogue—Child healthcare nurses’ lived experiences

**DOI:** 10.1111/scs.13072

**Published:** 2022-02-28

**Authors:** Ida Gustafsson, Malin Spångby, Ann Arvidsdal, Marie Golsäter, Lina Palmér

**Affiliations:** ^1^ 1802 Faculty of Caring Science, Work Life and Social Welfare University of Borås Borås Sweden; ^2^ Child Health Services Region Jönköping County Jönköping Sweden; ^3^ CHILD‐research Group School of Health and Welfare Jönköping University Jönköping Sweden; ^4^ Department of Health, Medicine and Caring Linköping University Linköping Sweden

**Keywords:** breastfeeding, breastfeeding difficulties, caring, caring dialogue, child healthcare nurses, existential, lifeworld, phenomenology, thematic analyze

## Abstract

Breastfeeding is experienced as an existential journey, and breastfeeding difficulties put mothers in existentially vulnerable situations. For care to be caring, it must be based on the mother's breastfeeding story. Previous research show that healthcare professionals struggle to perform individualised breastfeeding care. The Existential Breastfeeding Difficulty Scale (ExBreastS) was developed to support an existential focus in caring dialogues and was introduced in child healthcare in Sweden.

The aim of this study is to describe child healthcare nurses’ lived experience of how the Existential Breastfeeding Difficulty Scale (ExBreastS) influences the caring dialogue.

Seventeen child healthcare nurses with experience in using ExBreastS as a basis for caring dialogues with breastfeeding mothers were interviewed, in groups, pairs or individually. The interviews were analysed using a thematic analysis based on descriptive phenomenology.

The results show that the caring dialogue becomes re‐evaluated when using ExBreastS because existential aspects of breastfeeding is acknowledged. ExBreastS also visualises new perspectives of the mother's breastfeeding experiences. However, the use of ExBreastS also risks overshadowing the caring dialogue when the nurses focus too much on the instrument.

The use of ExBreastS supports caring dialogues—and caring care—by highlighting the existential aspects of breastfeeding/breastfeeding difficulties and the uniqueness of every mothers’ breastfeeding experience. However, the instrument sometimes evokes a vulnerability in the nurses that calls for support from the care organisation.

## INTRODUCTION

Initiating breastfeeding has been described as a collision between expectations and reality [1] and as an engrossing personal journey that requires individualised care and support [2]. Women's experiences of initiating breastfeeding tend to be overwhelming because they are attempting to learn how to breastfeed while physically recovering from birth [3]. Even fully functioning breastfeeding tends to be experienced as an existential challenge, and women often require support to trust in their own ability to breastfeed [4]. Additionally, the experience of severe breastfeeding difficulties has been associated with feelings of loneliness, suffering and a sense of existential lostness in motherhood [5]. Such an experience can awaken a sense of existential vulnerability, which will have negative effects on both the mother's relationship with the child and future breastfeeding [6]. Breastfeeding research show that up to 90% of new mothers reports initial breastfeeding difficulties [7, 8, 9], and such difficulties have been shown to be a major cause of early breastfeeding cessation [8]. Although there are biological aspects of breastfeeding to consider, breastfeeding care should also focus on the existential aspects of breastfeeding [10]. If healthcare professionals are sensitive to mothers’ unique needs, these mothers feel that the breastfeeding care is supportive in an individual and unique way [11]. However, mothers report that healthcare professionals tend not to show sufficient interest in or provide enough support to mothers with breastfeeding difficulties. In turn, mothers expect respect, empathy, sincerity, understanding, trust and assistance when having difficulties [12]. Previous research shows that healthcare professionals working with breastfeeding mothers strive to provide care that is sensitive to unique experiences and needs but that this is difficult to manage [11]. For breastfeeding care to be caring, a mother's unique breastfeeding story and cultural situatedness must be acknowledged in a caring dialogue [10].

To enable a caring dialogue with the breastfeeding story as a hub [10], the Existential Breastfeeding Difficulty Scale (ExBreastS) was developed and psychometrically tested in the context of Sweden [13]. ExBreastS is based on the results of phenomenological studies concerning the experience of initial breastfeeding difficulties [4, 5] and contains 16 items that focus on existential issues of breastfeeding, such as *I feel worthless at breastfeeding*, *breastfeeding has made the first time with my child hard to manage* and *breastfeeding makes me feel like a failure mother* [13]. The intention was to create a sensitive tool that healthcare professionals could use in caring dialogues to capture the existential aspects of experienced breastfeeding difficulties [13], regardless of the presence or degree of physical breastfeeding difficulties. Dialogues about breastfeeding already exist in the healthcare system, and there are instruments that can help healthcare professionals to care for breastfeeding mothers. However, the focus tends to be on biological and psychological factors, such as mothers’ or infants’ breastfeeding behaviour, mothers’ attitudes, knowledge and self‐efficacy or mothers’ satisfaction with breastfeeding [14, 15, 16]. From a caring science perspective, caring dialogue, or *caring conversation*, is essential to the caring relationship [17]. Due to the suffering and dependence of the patient, e.g. the mother, the caring dialogue is considered asymmetrical. The role and responsibility of the healthcare professional is to make space in which the narrative of the patient can unfold, giving the patient the opportunity to regain self‐confidence and wellbeing through self‐interpretation in a relationship of mutual vulnerability [18]. Caring can never be reduced to a specific technique or measure, but it is a health‐promoting action created in the encounter between healthcare professionals and the patient [19, 20]. The patient's perspective demands that healthcare professionals provide caring relationships and, in a caring dialogue, consider the lived experience and existential situation of each individual in order to strengthen health and wellbeing in existentially demanding situations [19]. Therefore, ExBreastS was introduced into clinical care to support child healthcare nurses in making the breastfeeding story visible through the caring dialogue. The aim of the study is to describe child healthcare nurses’ lived experience of how ExBreastS influences the caring dialogue with breastfeeding mothers.

## METHOD

### Introduction of ExBreastS in child healthcare in Sweden

In Sweden, child healthcare services have a responsibility to promote the child's health, wellbeing and development through care and support to the family and child from birth until preschool. Almost every mother and child attend scheduled visits, which are free of charge. One of the goals of child healthcare services is to promote and support breastfeeding [21]. In the present study, ExBreastS [13] was introduced in eight child healthcare units in a medium‐sized region in southern Sweden, prior to wider introduction and implementation in all child healthcare centres in the region. This was done in an ongoing project within women's and child healthcare intended to develop care according to a theoretical model that focusses on mothers’ breastfeeding stories as a basis for care [10]. The process of introduction consisted of forming and organising reflection groups for the child healthcare nurses before they began using ExBreastS. The nurses prepared by familiarise themselves with ExBreastS [13], as well as research about existential aspects of breastfeeding difficulties [5, 6, 22, 23]. The group reflections began after a short lecture. The reflections focussed on what it means to work as a child healthcare nurse caring for mothers with breastfeeding difficulties, how new knowledge about the existential aspects of breastfeeding may affect care and how ExBreastS can be used in caring dialogues. The nurses were instructed to use ExBreastS in caring dialogues for approximately 4 months. They were encouraged to use ExBreastS in dialogues with *all* mothers who were breastfeeding to some extent. This approach was chosen because the purpose of ExBreastS is to recognise mothers who *experience* breastfeeding difficulties, not just mothers who have objectively visible physical difficulties. The dialogues were conducted between 3 weeks and 4.5 months after birth. The mothers answered the questions either at home or at the child healthcare unit, and the answers were intended to serve as a basis for caring dialogues about breastfeeding.

### Participants

Seventeen child healthcare nurses with experience in using ExBreastS as a basis for caring dialogues about breastfeeding within the project participated in the study. The nurses were women between 34 and 64 years of age with a specialised training in either public health nursing or paediatric nursing. They had worked as nurses between 7 and 34 years and in child healthcare between 1.5 and 22 years. They worked in child healthcare units located in both urban and rural areas.

### Data collection

In the current study, open and pliable interviews were used for data collection [24]. Fourteen nurses participated in group interviews and were divided into three groups. Additionally, three nurses who were unable to attend the scheduled group interviews were interviewed, two in a pair interview and one in an individual interview. All the interviews began with the following open‐ended question: ‘Please tell us about your experiences of using ExBreastS in caring dialogues with breastfeeding mothers’. To deepen the descriptions, open questions were asked, including, for example, ‘Can you tell us more about that?’ ‘What does that mean to you?’ or ‘How did ExBreastS improve or hinder the caring dialogue about breastfeeding?’.

The interviews were conducted by MS and AA, but LP was involved in one group interview to provide feedback to the interviewees after the interview. All the interviews were performed at the nurses’ workplaces. The interviews lasted for up to 60 minutes and were audiotaped and transcribed verbatim.

### Analysis

The transcribed interviews were analysed using qualitative thematic analysis based on descriptive phenomenology [25]; this method focusses a phenomenon, by uncovering the meaning of lived experience. In this study, the phenomenon is *How ExBreastS influence the caring dialogue with breastfeeding mothers*, as experienced by child healthcare nurses. To describe meanings in data in a valid and rigorous way, the methodological principles of the thematic method guided the analysis. The principles emphasise openness, questioning one's preunderstanding and adopting a reflective attitude during the entire research process, especially during the analysis. The thematic analysis is described in three stages: achieve familiarity with the data through open‐minded reading, search for meanings, formulate themes based on meanings and organise themes into a meaningful wholeness [25].

The process of analysis began with an open‐minded reading, in which the interviews were read through several times in the initial familiarisation phase to obtain an overall view of the material, with a focus on how ExBreastS influences the caring dialogue with mothers who are breastfeeding. At this stage, the principle of openness was put into practice, with the intention of opening one's mind to the text and its meanings, as well as not letting one's preunderstanding overshadow new understandings. In the next stage, there was a moving back and forth between the whole (all interviews) and its parts (meanings) to search for the meanings connected to the aim. Meanings related to one another were compared to form patterns of meanings. During this process, there was a need to be open toward the data and one's own preunderstanding so as not to come to meanings too quickly. This was done by attempting to validate the patterns of meaning against the data through critical reflections on the part of the research team. In the last stage of the analysis, patterns of meanings were organised into three themes, which, together, formed a meaningful wholeness related to the aim. In this stage, again, critical reflections were made, and movement between the identified patterns of meanings and themes was performed to validate the themes.

### Ethical considerations

The current study was conducted in accordance with the requirements of the Helsinki Declaration [26]. Thus, the participants were treated based on the principles of autonomy, beneficence, nonmaleficence and justice. The participants received written and verbal information about the project and the study, in which confidentiality was assured and the possibility to withdraw at any stage of the research process without any responsibility toward the researcher was made clear. This type of study involving child healthcare nurses’ lived experience is not within the boundaries of the Ethics Review Act 2003:460, which regulates research involving humans in Sweden.

## RESULTS

**FIGURE 1:**
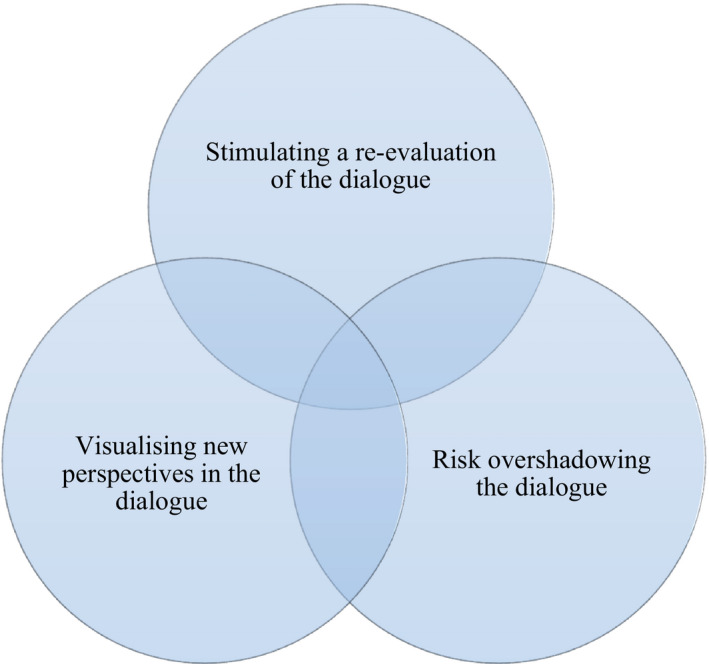
Themes of how ExBreastS influences the caring dialogue

The meaning of child healthcare nurses’ lived experience of how ExBreastS influences the caring dialogue with breastfeeding mothers is described through the following themes of meanings: *Stimulating a re*‐*evaluation of the dialogue*, *visualising new perspectives in the dialogue* and *risk overshadowing the dialogue* (Figure 1) The themes are described in the following sections, and quotes are used to show the lived experience and its meanings.

### Stimulating a re‐evaluation of the dialogue

The use of ExBreastS stimulates nurses to re‐evaluate the meaning of caring dialogues with breastfeeding mothers. Before the introduction of ExBreastS, the dialogues would tend to focus biologically and technically/practically questions. ExBreastS provides a space for the existential component of the breastfeeding experience, enriching the caring dialogues by illuminating the experiences of breastfeeding with or without breastfeeding difficulties as both a unique and complex phenomenon, as well as both an existential and biological one, making the importance of the dialogue more evident, as one nurse explains, ‘These deepest feelings, that you don't talk so much about, I think… No mother has actually told me about that kind of feelings [before using ExBreastS]’.

When the meaning of the caring dialogue is re‐evaluated, the woman's unique experiences are allowed to guide the dialogue. This helps the nurses realise that, when the dialogue does not consider the existential aspects of breastfeeding, the risk of taking for granted and/or misunderstanding how a woman experiences her breastfeeding situation is obvious. ExBreastS makes it clear that the experience of breastfeeding or breastfeeding difficulties does not have to align with the amount of physical breastfeeding difficulties or earlier breastfeeding experiences. One nurse states the following: ‘What surprised me, maybe, a little, was… that it was quite much anyway, that they had quite a lot of difficulties anyway’.

For the nurses, ExBreastS facilitates initiating dialogues about breastfeeding and breastfeeding difficulties that are normally experienced as difficult, private or delicate, without being perceived as judgemental. One nurse states the following: ‘You don't feel like the breastfeeding police if you ask these questions’. ExBreastS is also experienced as having the potential to help nurses notice hidden breastfeeding stories, for example, the stories of mothers who feel forced to breastfeed, even though they do not want to. One nurse states the following:

Maybe, they didn’t really want to breastfeed, but they do it. And I think you could be able to find them here, so to speak. [Now] that they, sort of, had the chance to express their feelings.

An awareness of the existential aspects of breastfeeding supports a new openness and responsiveness toward breastfeeding mothers. Working with ExBreastS not only changes the dialogue at hand but also causes nurses to re‐evaluate their entire way of thinking about the caring dialogue about breastfeeding. One nurse notes the following: ‘You have it with you in some way, even in other dialogues [in child healthcare]’. ExBreastS also makes nurses re‐evaluate the importance of continuous dialogue throughout the entire breastfeeding period. ExBreastS works like an icebreaker during the initial meeting, making the atmosphere of the dialogue more relaxed, which makes it easier to get the dialogue to flow. This facilitates future follow‐up dialogue, as one nurse says, ‘It can be easier for us to find the words when we follow it up [the breastfeeding] that “when we met in the beginning, when you completed ExBreastS, then you said this or that — how do you feel about that today?”’.

However, the re‐evaluation of the importance of an in‐depth caring dialogue with a focus on the existential aspects of breastfeeding and breastfeeding difficulties illuminates the fact that the reality of child healthcare—with its time constraints and several other instruments that must be used—does not fully support these kinds of dialogues. One nurse says, ‘I think that, if you shall fill it in, then I shall have the time to look at the results as well’.

### Visualising new perspectives in the dialogue

In the caring dialogues following ExBreastS, the nurses experience that the gap between the mothers’ expectations of breastfeeding and the reality can be visualised, which gives the mothers a chance to view themselves and their situation from a new perspective. The nurses also experience that ExBreastS helps them to obtain a more realistic understanding of the mothers’ breastfeeding experiences. This is true regardless of the degree of physical or experienced breastfeeding difficulties. One nurse says, ‘And it is okay to feel like that… even if I [the mother] have a good breastfeeding experience, I don't always need to think that it is fantastic’. According to the nurses, ExBreastS may also make mothers aware of the continuous process of the breastfeeding experience. One nurse states the following: ‘Then, she could see that, now, it has improved because “I felt like this ten days ago, and now, it's a bit different”’.

The nurses experience that the existential questions in ExBreastS—and the following caring dialogues—provide an opportunity for the mothers to put words to their experiences and have the potential to legitimate thoughts and feelings that the mothers may otherwise think are unique to them. One nurse recounts a dialogue in which a woman expresses this feeling: ‘This question exists. Then, there must be somebody else who has felt like this’.

However, the nurses express their fear that ExBreastS could foster negative feelings about breastfeeding among mothers on the edge of stopping breastfeeding. There is also a perceived risk that mothers with no former negative breastfeeding experience will not recognise themselves in the existential aspects of breastfeeding represented in ExBreastS. On the contrary, the nurses experience that some mothers could be helped to see that they are actually privileged in not having severe breastfeeding difficulties and/or negative breastfeeding experiences. One nurse notes the following: ‘They reflected a lot about that they felt gratitude because it actually worked that well’.

### Risk overshadowing the dialogue

Although ExBreastS is experienced as having a mainly positive impact on the caring dialogue, the nurses experience that a too extensive focus on ExBreastS may overshadow the caring dialogue. In some situations, ExBreastS can even be experienced as redundant because, to some extent, the care already includes a dialogue with the capacity to address the existential aspects of the breastfeeding experience; here, then, the instrument takes time away from the actual dialogue. A nurse explains the following: ‘I would have got the same answers from her anyway, without this instrument, actually …it was nothing that made me surprised’.

Sometimes, practical difficulties connected with the design of ExBreastS may overshadow the dialogue. The nurses ask for an easier way to obtain a quick overview of the answers in ExBreastS, which would help focus on the dialogue instead of the instrument itself. The nurses also experience that ExBreastS must be simple and clear to ensure that the women respond to it. Too many questions or confusing alternatives may overshadow the dialogue. ExBreastS is also experienced as quite open to interpretation—for better or worse. A question charged with negativity could be interpreted in a positive way, which may create confusion in the dialogue. One nurse states the following:

And I said to her that, if you breastfeed once an hour and sit there, of course, you feel trapped, but she [the mother] thought… she understood, of course, that this feeling was supposed to be something negative, but for her, it was more like ‘this is how it is’.

Even if ExBreastS affects all dialogues—regardless of whether it is used directly—mothers with limited language skills do not have the same chance of being invited to these in‐depth existential dialogues. Here, the caring dialogue about breastfeeding may be affected in a negative way. However, being a native speaker does not ensure one will fully understand the questions in ExBreastS. When there is confusion about the meaning of the questions, the dialogue can easily become overshadowed, as one nurse explains, ‘It can almost be a little hard to explain yourself too… Because I also thought, “What is it really?”’ Sometimes, the nurse's uncertainty overshadows the dialogue, creating a desire to protect the mothers from harm by not handing the instrument out to those experienced as especially sensitive to the content of the instrument.

## DISCUSSION

The results of the current study are novel and unique because instruments considering mothers’ lived experience of breastfeeding and breastfeeding difficulties, such as ExBreastS, are lacking in both clinical settings and previous research. Additionally, studies that introduce or implement specific caring activities are rare, and this is the first time that ExBreastS has been introduced and evaluated in a clinical setting. In this study, the aim was to describe child healthcare nurses’ lived experience of how ExBreastS influences the caring dialogue with breastfeeding mothers. An evaluation of the usability of and potential for improvement in the instrument is to be presented elsewhere. As stated in the background, most of the instruments used in clinical settings focus on the objective, physical and/or psychological issues of breastfeeding and/or breastfeeding difficulties [14, 15, 16]. According to the results of the current study, ExBreastS has the potential to fill a gap in caring practice, one described by both mothers and healthcare professionals as a need for sensitivity to experiences of breastfeeding and breastfeeding difficulties [4, 5, 11, 12, 27, 28, 29]. The results of our study show that, when ExBreastS is used in the dialogue, child healthcare nurses are surprised at what mothers report regarding the existentiality of breastfeeding, partly because the experience, obviously, need not correspond to the physical or practical difficulties of breastfeeding. This is interesting because, before the use of ExBreastS, there is a risk that mothers have not been confirmed in their existential situation due to a too excessive focus on physical breastfeeding difficulties. From a caring science perspective [19, 20], it is important to intertwine the physical/objective and existential/subjective aspects of care. Listening to a mother's breastfeeding story has been described as one way to approach the existential aspects of breastfeeding and provide care that is caring [10]. Granberg et al. [30] show that mothers’ subjective experiences of breastfeeding, in terms of a well‐functioning relationship with the child, are an important factor in the enjoyment of breastfeeding. This demonstrates the need to use ExBreastS to re‐evaluate and visualise the existential perspectives of the caring dialogue.

After the introduction of ExBreastS, some nurses expressed a desire for an instrument with fewer interpretation options, one that could be evaluated at a glance. It is possible that a partial explanation for this desire was time pressure. This is visible in the results, in which a nurse expresses that one prerequisite for ExBreastS to be caring is sufficient time for the dialogue. The experience of time pressure is consistent with previous research on nurses’ working conditions [31] and demonstrates the need for a reprioritisation of care organisation in order to provide space for caring dialogues. Another explanation for the desire for efficiency could be the child healthcare nurses’ previous experience of using screening instruments with simple scoring in their daily work [32]. Thus, if ExBreastS is to be implemented in a broader context, there seems to be a need to clarify the purpose of the instrument as a support for caring dialogues, as opposed to a screening tool. From a caring dialogue point of view, it may even be an advantage that the questions are open to interpretation because caring dialogues should allow for individuality and self‐interpretation [18]. Of course, this only applies to the point that the questions are considered understandable. Apart from this, the use of ExBreastS seems to be a feasible way to develop child healthcare nurses’ skills and attention to having a caring dialogue about the existential aspects of breastfeeding and breastfeeding difficulties. The results show that the introduction and use of ExBreastS make nurses more sensitive to the existential aspects of breastfeeding, even if the instrument is not used in the current dialogue. In this way, ExBreastS has the potential to inspire dialogues with an existential focus in other areas of child healthcare as well.

Introducing caring dialogues with an existential focus, based on ExBreastS, as a way of approaching breastfeeding and breastfeeding difficulties in child healthcare in the context of Sweden is—according to the results of the current study—both demanding and relieving for the child healthcare nurses. The demands manifest themselves in feelings of uncertainty, uneasiness and insecurity in the new caring role. This could be interpreted as an evoked vulnerability. Previous research confirms the interpretation of vulnerability by showing that nurses own existential concerns are awakened by existential issues in care [33]. Nurses and physicians working at neonatal intensive care have expressed difficulties in dealing with parents’ existential issues [34], and existential care has been shown to make nurses feel insecure about meeting and adequately respond to patients’ existential needs [35]. It is relevant to reflect on the possibility that nurses experiencing this uncertainty are not open to an existential dialogue, despite the instrument, and may be in need of support from the care organisation. On the other hand, Galvin and Todres [36] state that the vulnerability evoked in a caring dialogue is a necessity for the dialogue to be caring and that being touched is a basis for humanising care [18]. This indicates a need for child healthcare nurses to have the courage to dwell in the caring dialogue despite feelings of vulnerability.

One current discussion in Sweden concerns the need for better continuity between the various components of care during the childbearing period. Previous research [37] highlights the fact that continuity in breastfeeding support counselling has the potential to increase experiences of coherence in the care of breastfeeding mothers. This kind of support has made mothers more satisfied and positively influenced mothers’ abilities to handle breastfeeding difficulties while also increasing breastfeeding duration [37]. From the mother's perspective, healthcare professionals working to support breastfeeding mothers must offer sensitive, individualised breastfeeding support to promote a positive breastfeeding experience. Creating a respectful and mutual dialogue is one way of doing this [27]. This is in line with what is suggested in a theoretical model of caring for mothers with breastfeeding difficulties [10]. Based on the results of the current study, ExBreastS has the potential to complement other types of breastfeeding support and—together with the breastfeeding story [10]—identify and care for women who struggle with the existential aspects of breastfeeding. Further research is needed on mothers/parents’ experiences of how ExBreastS influences the caring dialogue and the instrument needs to be implemented and evaluated in other parts of the care chain (primarily maternal healthcare) and in midwifery and child healthcare education. In this way, ExBreastS has the potential to help fill the gap in care continuity by providing a foundation for the caring dialogue before, during and after the actual breastfeeding period.

## METHODOLOGICAL CONSIDERATIONS

A qualitative thematic analysis based on descriptive phenomenology [25] was used to analyse the results. This was considered a suitable method for describing the lived experience as perceived by child healthcare nurses, which contributed to the validity of the study. One limitation of the study could be that the nurses participated in different kinds of interviews (group, pair and individual), which could have affected the results. On the other hand, ethically, it was important to be sensitive to the nurses’ unique prerequisites and expressed needs due to heavy workloads or travel distance in relation to voluntary participation. Individual and group interviews have also been shown to complement and nuance one another [38], which indicates that the varied types of interviews could be seen as a strength. Another methodological limitation could be that the nurses had a varied amount of experience using the instrument, in which some nurses had only tested the instrument a few times (e.g. because of ambiguities in considering which mothers were suitable for participation). Overall, the nurses had used ExBreastS to that extent that they had various experiences with it to express, which strengthens the results. Another strength is the careful introduction of ExBreastS in terms of lecturing and reflection (LP, MG) that the nurses took part in before the use of ExBreastS, as well as the nurses’ enthusiasm and willingness to participate. On the other hand, one limitation is that the nurses participating in the study did not receive any organisational benefits, such as extra time for the caring dialogue. For some of the nurses, this prerequisite negatively influenced their participation in the study because they did not experience that they were provided with sufficient conditions in which to implement the caring dialogues. A further limitation could be the fact that the interviewers had no previous experience in conducting qualitative interviews. On the other hand, the research group, as a whole, has extensive experience with qualitative interviews and analysis. The questions used during the interviews, as well as the interviews themselves, were discussed within the research group. One of the experienced co‐authors participated during the first interview to provide feedback, and the authors conducting the interviews took turns taking notes as the interviews were being conducted. Ultimately, the transcribed interviews turned out to be rich in meaning, strengthening the quality of the interviews. To further ensure the quality of the interviews, the authors conducting the interviews (MS and AA) and carrying out the main component of the analysis (IG) did not take part in the process of developing (LP) or introducing the instrument (LP and MG). Scientific rigour was further ensured by being true to the core concepts of the chosen method—openness, questioning preunderstandings and having a reflective attitude [25]—throughout the entire research process. The process of analysis has been allowed to take time to increase the reflexivity of the study [25]. The usefulness and relevance of the study results may depend on the context [25]. The current study was performed in Sweden. Child healthcare nurses around the world may have varied knowledge and prerequisites in relation to breastfeeding care. With this in mind, the results are considered transferable to clinical settings that are similar to the Swedish context.

## CONCLUSIONS

The use of ExBreastS in the context of child healthcare in Sweden supports caring dialogues by highlighting the existential aspects of breastfeeding and breastfeeding difficulties. The instrument visualises the uniqueness of every breastfeeding experience, providing conditions for continuous and individualised caring. For different reasons it is sometimes demanding for the nurses to conduct dialogues about existential issues, which places them in a vulnerable situation. Therefore, a wider implementation of ExBreastS requires a care organisation that offers support to strengthen nurses’ ability to talk and reflect about the existential aspects of breastfeeding and breastfeeding difficulties, as well as allowing the caring dialogue sufficient time to unfold.

## CONFLICT OF INTEREST

The authors declare no potential conflicts of interest regarding the research, authorship and/or publication of the article.

## AUTHOR CONTRIBUTION

LP, MG, IG, MS and AA were involved in the study design. LP and MG were involved in introducing the ExBreastS in clinical settings. LP, MS and AA collected data. LP, MG, IG, MS and AA analyzed the data. LP and IG drafted the manuscript, and LP, MG and IG revised it.

## References

[1] Burns E , Schmied V , Sheehan A , Fenwick J . A meta‐ethnographic synthesis of women’s experience of breastfeeding. Matern Child Nutr. 2010;6(3):201–19. 20929493 10.1111/j.1740-8709.2009.00209.x PMC6860551

[2] Nelson AM . A metasynthesis of qualitative breastfeeding studies. J Midwifery Womens Health. 2006;51(2):e13–20. 16504899 10.1016/j.jmwh.2005.09.011

[3] Sheeran L , Buchanan K , Welch A , Jones LK . Women’s experiences of learning to breastfeed. Breastfeed Rev. 2015;23(3):15–22. 27183770

[4] Palmér L , Carlsson G , Mollberg M , Nyström M . Breastfeeding: an existential challenge – women’s lived experiences of initiating breastfeeding within the context of early home discharge in Sweden. Int J Qual Stud Health Wellbeing. 2010;5(3):1–11. 10.3402/qhw.v5i3.5397 PMC2964113 20978548

[5] Palmér L , Carlsson G , Mollberg M , Nyström M . Severe breastfeeding difficulties: existential lostness as a mother. Women’s lived experiences of initiating breastfeeding under severe difficulties. Int J Qual Stud Health Wellbeing. 2012;7. 10.3402/qhw.v7i0.10846 PMC3272819 22312409

[6] Palmér L , Carlsson G , Brunt D , Nyström M . Existential vulnerability can be evoked by severe difficulties with initial breastfeeding: a lifeworld hermeneutical single case study for research on complex breastfeeding phenomena. Breastfeed Rev. 2014;22(3):21–32. 25522459

[7] Chaput K , Nettel‐Aguirre A , Musto R , Adair C , Tough S . Breastfeeding difficulties and supports and risk of postpartum depression in a cohort of women who have given birth in Calgary: a prospective cohort study. CMAJ Open. 2016;4(1):E103–9. 10.9778/cmajo.20150009 PMC4866929 27280109

[8] Almgren Tangen G , Bergman S , Dahlgren J , Roswall J , Alm B . Factors associated with discontinuation of breastfeeding before 1 month of age. Acta Paediatr. 2012;101(1):55–60. 21767302 10.1111/j.1651-2227.2011.02405.x

[9] Gianni ML , Bettinelli ME , Manfra P , Sorrentino G , Bezze E , Plevani L , et al. Breastfeeding difficulties and risk for early breastfeeding cessation. Nutrients. 2019;11(10):2266. 10.3390/nu11102266 PMC6835226 31547061

[10] Palmér L , Gustafsson I . A theoretical model on caring for mothers with initial breastfeeding difficulties: the breastfeeding story as a hub for caring practice. Int J Hum Caring. 2021;25(1):45–59.

[11] Bäckström C , Hertfelt Wahn E , Ekström A . Two sides of breastfeeding support. Experiences of women and midwives. Int Breastfeed J. 2010;5(20):1–20. 21114812 10.1186/1746-4358-5-20 PMC3001698

[12] Durmazoğlu G , Yenal K , Okumuş H . Maternal emotions and experiences of mothers who had breastfeeding problems: a qualitative study. Res Theory Nurs Pract. 2020;34(1):3–20. 31937633 10.1891/1541-6577.34.1.3

[13] Palmér L , Jutengren G . Development and psychometric testing of an instrument to assess existential aspects of mother's initial breastfeeding difficulties (ExBreastS). Sex Reprod Healthc. 2019;19:88–94. 30928141 10.1016/j.srhc.2019.01.005

[14] Dennis C , Faux S . Development and psychometric testing of the Breastfeeding Self‐Efficacy Scale. Res Nurs Health. 1999;22(5):399–409. 10520192 10.1002/(sici)1098-240x(199910)22:5<399::aid-nur6>3.0.co;2-4

[15] Leff E , Jeffries S , Gagne M . The development of the Maternal Breastfeeding Evaluation Scale. J Hum Lact. 1994;10(2):105–11. 7619250 10.1177/089033449401000217

[16] Jensen D , Wallace S , Kelsay P . LATCH: a breastfeeding charting system and documentation tool. J Obstet Gynecol Neonatal Nurs. 1994;23(1):27–32. 10.1111/j.1552-6909.1994.tb01847.x 8176525

[17] Arman M , Ranheim A , Rydenlund K , Rytterström P , Rehnsfeldt A . The Nordic tradition of caring science: the works of three theorists. Nurs Sci Q. 2015;28(4):288–96. 26396212 10.1177/0894318415599220

[18] Fredriksson L , Eriksson K . The ethics of the caring conversation. Nurs Ethics. 2003;10(2):138–48. 12659485 10.1191/0969733003ne588oa

[19] Dahlberg K , Segesten K . Hälsa och vårdande: I teori och praxis (Health and caring: In theory and practice). Stockholm: Natur & Kultur; 2010.

[20] Dahlberg K . Lifeworld Phenomenology for Caring and Health Care Research. London: Routledge; 2011.

[21] Socialstyrelsen . Vägledning för barnhälsovården [the National Board of Health and Welfare. Guidance for child health care]. Falun: Sweden: Edita Bobergs; 2014.

[22] Palmér L , Carlsson G , Brunt D , Nyström M . Existential security is a necessary condition for continued breastfeeding despite severe initial difficulties: a lifeworld hermeneutical study. Int Breastfeed J. 2015;10(17):1–12. 25960763 10.1186/s13006-015-0042-9 PMC4425864

[23] Palmér L . Previous breastfeeding difficulties: an existential breastfeeding trauma with two intertwined pathways for future breastfeeding — fear and longing. Int J Qual Stud Health Wellbeing. 2019;14(1):1588034. 10.1080/17482631.2019.1588034 PMC6442107 30893016

[24] Kvale S , Brinkmann S . Den kvalitativa forskningsintervjun (The qualitative research interview). Lund: Studentlitteratur; 2014.

[25] Sundler AJ , Lindberg E , Nilsson C , Palmér L . Qualitative thematic analysis based on descriptive phenomenology. Nurs Open. 2019;6(3):733–9. 31367394 10.1002/nop2.275 PMC6650661

[26] World Medical Association (WMA) . WMA Declaration of Helsinki. Ethical principles for medical research involving human subjects. Revised; 2013. 10.1001/jama.2013.281053 24141714

[27] Blixt I , Johansson M , Hildingsson I , Papoutsi Z , Rubertsson C . Women’s advice to healthcare professionals regarding breastfeeding: “offer sensitive individualized breastfeeding support” – an interview study. Int Breastfeed J. 2019;14(51):1–12. 31889974 10.1186/s13006-019-0247-4 PMC6916109

[28] Gustafsson I , Nyström M , Palmér L . Midwives’ lived experience of care for new mothers with initial breastfeeding difficulties: a phenomenological study. Sex Reprod Healthc. 2017;12:9–15. 28477939 10.1016/j.srhc.2016.12.003

[29] Debevec AD , Evanson TA . Improving breastfeeding support by understanding women's perspectives and emotional experiences of breastfeeding. Nurs Womens Health. 2016;20(5):464–74. 27719776 10.1016/j.nwh.2016.08.008

[30] Granberg A , Ekström‐Bergström A , Bäckström C . First‐time mothers’ enjoyment of breastfeeding correlates with duration of breastfeeding, sense of coherence, and parental couple and child relation: a longitudinal Swedish cohort study. Nurs Res Pract. 2020;2020:8194389. 32637175 10.1155/2020/8194389 PMC7321520

[31] Olofsson B , Bengtsson C , Brink E . Absence of response: a study of nurses’ experience of stress in the workplace. J Nurs Manag. 2003;11(5):351–8. 12930542 10.1046/j.1365-2834.2003.00384.x

[32] Cox JL , Holden JM , Sagovsky R . Detection of postnatal depression. Development of the 10‐item Edinburgh Postnatal Depression Scale. Br J Psychiatry. 1987;150:782–6. 3651732 10.1192/bjp.150.6.782

[33] Lundvall M , Lindberg E , Hörberg U , Palmér L , Carlsson G . Healthcare professionals’ lived experiences of conversations with young adults expressing existential concerns. Scand J Caring Sci. 2019;33(1):136–43. 30152541 10.1111/scs.12612

[34] Wigert H , Bry K . Dealing with parents’ existential issues in neonatal intensive care. J Neonatal Nurs. 2018;24(4):213–7.

[35] Prause D , Sørlie V , Danbolt LJ , Tornøe K . Sensing loneliness – Nurses’ experiences with providing existential care to older patients with acquired deafblindness. Nordisk Sygeplejeforskning. 2020;10(4):280–91.

[36] Galvin K , Todres L . Caring and Well‐being. A Lifeworld Approach. London, UK: Routledge: Taylor & Francis Group.

[37] Blixt I , Martensson LB , Ekström AC . Process‐oriented training in breastfeeding for health professionals decreases women’s experiences of breastfeeding challenges. Int Breastfeed J. 2014;9(15):1–9. 25221613 10.1186/1746-4358-9-15 PMC4163059

[38] Lambert SD , Loiselle CG . Combining individual interviews and focus groups to enhance data richness. J Adv Nurs. 2008;62:228–37. 18394035 10.1111/j.1365-2648.2007.04559.x

